# Supplementary figures and images for: An integrated modeling, verification, and code generation for uncrewed aerial systems: less cost and more efficiency
Source: PeerJ Comput Sci. 2025 Jan 9;11:e2575. doi: 10.7717/peerj-cs.2575 (PMC11784798; doi:10.7717/peerj-cs.2575)

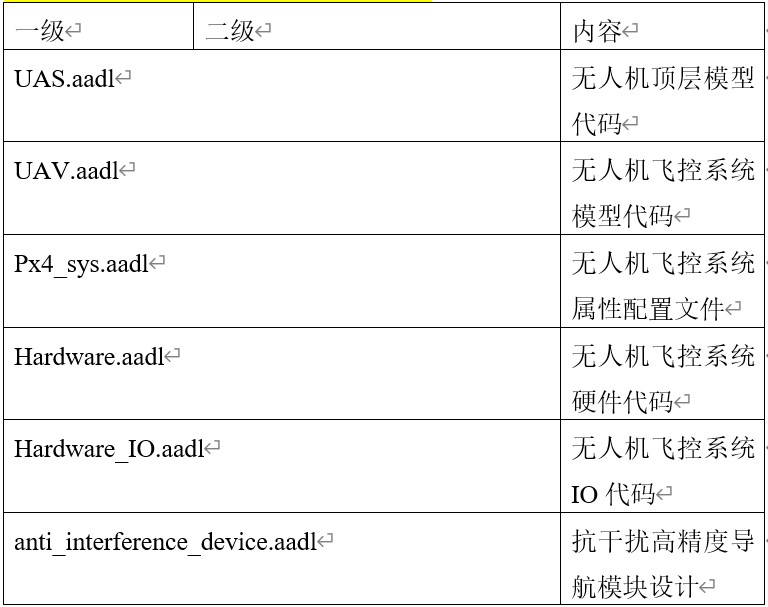

Supplement: Supplemental Information 1 [file peerj-cs-11-2575-s001.zip › ┤·┬δ/┤·┬δ╬─╝■╜Θ╔▄-1.jpg]

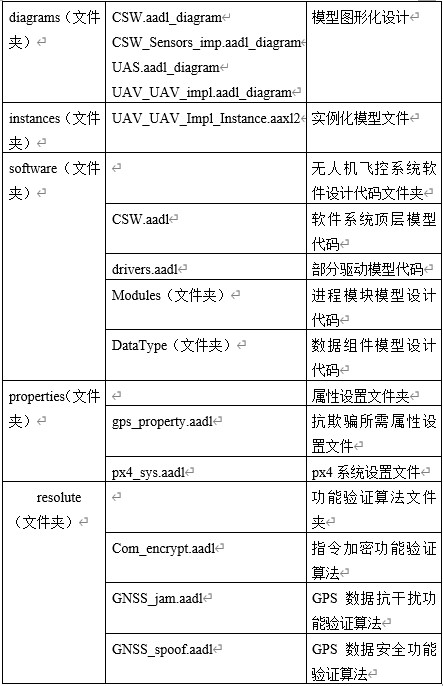

Supplement: Supplemental Information 1 [file peerj-cs-11-2575-s001.zip › ┤·┬δ/┤·┬δ╬─╝■╜Θ╔▄-2.jpg]
